# Exosomes derived from mesenchymal stem cells in diabetes and diabetic complications

**DOI:** 10.1038/s41419-024-06659-w

**Published:** 2024-04-17

**Authors:** Yu-Rui Jiao, Kai-Xuan Chen, Xiang Tang, Yu-Long Tang, Hai-Lin Yang, Yu-Long Yin, Chang-Jun Li

**Affiliations:** 1 Department of Endocrinology, Endocrinology Research Center, Xiangya Hospital, Central South University, Changsha, Hunan 410008 China; 2 Key Laboratory of Agro-ecological Processes in Subtropical Region, Institute of Subtropical Agriculture, Chinese Academy of Sciences, Changsha, Hunan 410125 China; 3 Department of Orthopaedics, The Second Affiliated Hospital of Fuyang Normal University, Fuyang, Anhui 236000 China; 4 College of Animal Science and Technology, Hunan Agricultural University, Changsha, Hunan 410128 China; 5 Key Laboratory of Aging-related Bone and Joint Diseases Prevention and Treatment, Ministry of Education, Xiangya Hospital, Central South University, Changsha, Hunan 410008 China; 6 National Clinical Research Center for Geriatric Disorders, Xiangya Hospital, Central South University, Changsha, Hunan 410008 China; 7 Laboratory Animal Center, Xiangya Hospital, Central South University, Changsha, Hunan 410008 China

**Keywords:** Mesenchymal stem cells, Diabetes complications

## Abstract

Diabetes, a group of metabolic disorders, constitutes an important global health problem. Diabetes and its complications place a heavy financial strain on both patients and the global healthcare establishment. The lack of effective treatments contributes to this pessimistic situation and negative outlook. Exosomes released from mesenchymal stromal cells (MSCs) have emerged as the most likely new breakthrough and advancement in treating of diabetes and diabetes‐associated complication due to its capacity of intercellular communication, modulating the local microenvironment, and regulating cellular processes. In the present review, we briefly outlined the properties of MSCs-derived exosomes, provided a thorough summary of their biological functions and potential uses in diabetes and its related complications.

## Facts

- The biological significance of mesenchymal stem cells derived exosomes (MSCs-Exos) has been deeply studied and has greatly developed in treating of diabetes and diabetes‐associated complication.
- The information transmission function of MSCs-Exos makes it important in maintaining β-cells homeostasis and insulin sensibility.
- Several crucial aspects of MSCs-Exos still need to be taken into account about preclinical experiments, including the determination of the optimal tissue source of MSCs-Exos, the migratory capabilities to specific sites, and the optimization of administration routes.

## Open questions

- How do transplanted MSCs-Exos influence crosstalk within the islets microenvironment?
- How to maximize the efficiency of MSCs-Exos-based therapy?
- How to dissolve problems faced by the clinical transformation of MSCs-Exos in cell-free therapy?

## Introduction

Diabetes mellitus (DM), a collection of metabolic syndromes characterized by long-term hyperglycemia, is a prevalent public health problem with complex etiology. By 2045, its incidence is expected to rise to 693 million [1]. Persistent hyperglycemia may induce the establishment of diabetes‐associated complications, such as diabetic kidney disease (DKD), diabetic cardiomyopathy (DCM), diabetic neuropathy, diabetic retinopathy (DR), diabetic wounds and diabetic foot ulcers [2]. Diabetes and its complications have contributed tremendously to the disability and mortality and the burden of economic worldwide [2, 3]. Numerous hypoglycemic drugs which aim to reducing or postponing the complication are available clinically, yet effective therapies to reverse DM-related organ damage are lacking [2]. For this reason, there exists a pressing imperative to find and develop practical alternative approaches or drug molecules to prevent and delay the complications of DM.

MSCs possess the capacity for undergoing self-renewal and the generation of specific differentiated cells, and are gaining interest as a novel cell-based therapy ageinst diabetes [4, 5]. MSCs may be derived from multiple sources including bone marrow, adipose, umbilical cord, menstrual blood and placenta. MSCs have emerged as the most promising and suitable source of cells for transplantation due to their low immunogenicity. Nevertheless, there are limitations in MSCs therapy, namely organ residence, the risk of microthrombosis, cellular rejection by the recipient, and tumorigenicity potential [6]. Researchers demonstrated that MSCs provide the therapeutic benefits mostly through paracrine actions [7]. Mesenchymal stem cell-derived exosomes (MSCs-Exos) have demonstrated comparable efficacy to MSCs in the treatment of diabetes and its associated complications [8]. MSCs-Exos have shown a superior therapeutic and regenerative effect in treating DM compared with the original cells in some research investigations [8]. Furthermore, Exosomes are easier to maintain than MSCs and are considered a safer option due to their lower amount of membrane-bound proteins and absence of direct tumorigenicity [9]. Cell-free exosome therapy is a novel approach for the treatment of diabetes and diabetic complications since it can circumvent the aforementioned drawbacks of MSCs transplantation while still having excellent therapeutic effects and safety.

In this review, we summarized the biological characteristics of exosomes and outlined the updated understanding about the utilization of MSCs derived exosomes as a therapeutic approach for DM and its complications. This review also explained the underlying potential mechanisms and elucidated the current state-of-the-art advances in preclinical studies about MSCs-Exos.

## Mesenchymal stem cells derived exosomes

### Origin

Exosomes, a group of nanoparticles ranging of 30–150 nm in diameter, were first discovered by Pan and Johnstone in 1983 [10]. Exosomes are generated by the invagination of the membrane of early endosomes, originated from multivesicular bodies (MVBs) [11]. Specifically, early endosomes were generated by inward budding of the plasma membrane. Subsequently, exosomal vesicles were formed by budding into the limiting membrane of early endosomes. A portion of RNAs and proteins were packed into the intraluminal vesicles through the use of either endosomal sorting complex required for transport dependent (ESCRT) machinery or ESCRT-independent machinery. The endosomes engulf part of the cytoplasm and give rise to an MVB [11, 12]. Exosomes biogenesis requires necessitates the involvement of four multiprotein sub-complexes, namely ESCRT-0 and ESCRT-I, -II, and -III, which collectively constitute the ESCRT machinery. The initial ESCRT complexe and ubiquitinated proteins are bound together, resulting in the formation of protein complexes in the cytoplasm [13].

### Characteristics

MSCs, a kind of stem cell with multipotent capabilities, may be located in many organs such as bone marrow and adipose. MSCs have the ability to differentiate into several cell types, including osteoblasts, adipocytes, chondroblasts and myocytes. MSCs-derived exosomes participate in intercellular communication by facilitating the transfer of various molecules including proteins, mRNAs, long noncoding RNAs, miRNAs, and metabolic enzymes into targeted cells [14]. Additional molecules, including tetraspanins (e.g., CD9, CD81, and CD63), heat shock proteins (HSP 70 or 90), and integrins, can also be packaged into exosomes, which play a role in assembly or intracellular trafficking [11]. Furthermore, Exosomes derived from MSCs are also perfect delivery systems for compounds like therapeutic genes, medicines, enzymes, or RNA to reach specific cells in addition to their inherent qualities [15].MSCs-Exos have been shown to possess the ability to safeguard their contents against destruction and and facilitate their intracellular uptake via endocytosis [16]. Additionally, MSCs-Exos offer a range of advantages over MSCs, including reduced immunogenicity, enhanced stability, and simplified storage [9]. Exosomes can initiate various pathophysiological responses, such as cell proliferation, reproduction and development, immunological regulation, homeostasis, and neural communication in the recipient cells by interacting with receptors and mediating signal transduction pathways [17]. Multiple studies have demonstrated that MSCs-Exos, as a viable alternative to MSCs, possess comparable functionalities to MSCs. Recently, exosomes have garnered growing interest as an alternate option of cell-free to the existing available stem cell therapies.

### MSCs-exos heterogeneity

The heterogeneity of MSCs-Exos may be conceptualized on the basis of their size, content (cargo), and particularly cellular origin (source) [11]. Exosomes secreted by MSCs of different tissues carry different biological components that confer unique biological roles to the exosomes [18]. BMSCs are the earliest MSCs isolated and obtained, and are the most studied and applied type of MSCs in stem cell therapy [19]. The safety and efficacy of BMSCs derived Exos have been confirmed in several studies. BMSCs are considered the optimal seed cells for isolating exosomes due to their rapid proliferation rate and the ease of in vitro isolation and culture [20]. ADMSCs are MSCs that are abundantly stocked in the human body. They possess high proliferation and differentiation capabilities, exhibit immunomodulatory effects, and are easily accessible and cost-effective. Adipose tissue contains a larger concentration of MSCs compared to bone marrow and other sources. Obtaining exosomes from adipose stem cells is less invasive and lacks ethical limits when compared to bone marrow stem cells, as well as immunomodulatory properties [21]. Additionally, UCMSCs derived exosomes are extracted from discarded umbilical cords. UCMSCs are cost-effective, less invasive, easily isolatable, highly self-renewing capacity, more effective in gene transfection, and ethically preferable source of Exos [22]. Since it lacks antigens associated with transplant rejection, it is appropriate for transplantation between different individuals. Meanwhile, the bone marrow required for BMSCs-Exos may come from individuals of different ages, which has a greater impact on exosomes biological activity, whereas the characteristics of UCMSCs-Exos do not have this problem. Similarly, MenSCs-Exos exhibit more proliferative capacity than BMSCs because they are easily obtained non-invasively and are plentiful [23]. Uniquely, gingival mesenchymal stem cells are highly proliferative and have the propensity to differentiate into neural lineage cells due to the neural crest-origin. GMSC-derived exosomes exhibit comparable biological functions and therapeutic effects as GMSCs, thus representing a promising option for cell-free therapy [24].

Exosomes can reflect their cellular origin and the physiological state of the cells, therefore MSCs-Exos from different sources have different characteristics and functions. Exploring the characteristics and functions of exosomes from different cell sources is expected to provide new means for drug delivery systems and precision therapy.

## MSCs derived exosomes in the maintenance of blood glucose homeostasis

The pancreatic β-cells are the exclusive producers that produce and coordinators of insulin hormone secretion, which plays a pivotal function in the regulation of glucose homeostasis. β-cells dysfunction is a crucial factor in the advancement of both type 1 diabetes mellitus (T1DM) and type 2 diabetes mellitus (T2DM), which are intricate and heterogeneous diseases with the common consequence—blood glucose levels (hyperglycemia) [25]. According to the American Diabetes Association, T1DM is defined as autoimmune β-cells destruction and death, typically resulting in absolute insulin deficiency, and T2DM is distinguished by a persistent reduction of β-cells mass loss and a progressive loss of insulin secretion. Both conditions are accompanied by peripheral insulin resistance. Therefore, to reverse β-cells harm and even to facilitate the regeneration of β-cells are the promising and ultimate aims of treating insulin-dependent diabetes.

**Fig. 1:**
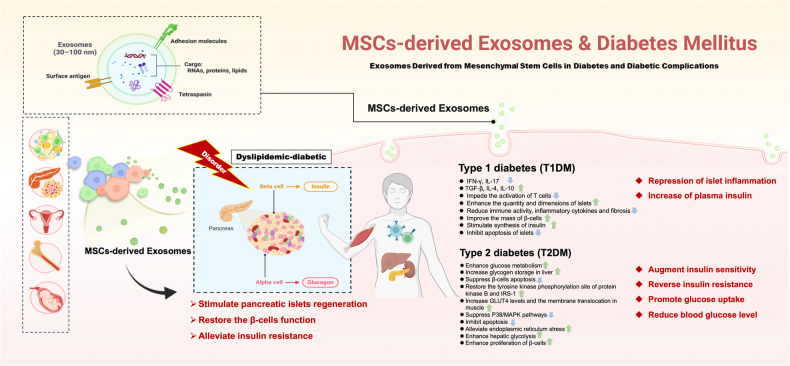
The involvement of exosomes derived from MSCs in the pathogenesis of diabetes mellitus. This critical function includes stimulation of islet regeneration, restoration of β-cell function, and attenuation of insulin resistance. Specifically, MSCs-derived exosomes can inhibit islet inflammation and increase plasma insulin levels in T1DM, enhance insulin sensitivity, reverse insulin resistance, promote glucose uptake, and reduce blood glucose levels in T2DM. Green arrows indicate up-regulation and blue arrows represent down-regulation.

Diabetes is becoming increasingly prevalent globally, with an estimated 4.2 million adults aged 20–79 fatalities attributed to diabetes in 2019, representing 11.3% of all causes of death [26]. Diabetes and its associated consequences are recognized as a substantial contributor to the rise in morbidity and mortality worldwide [2]. Controlling the symptoms of DM often involves the utilization of insulin pump or daily insulin injections, as well as the administration of pharmacological agents such metformin, thiazolidinedione, and sulfonylurea, for the duration of the patient’s life [27]. Nevertheless, it should be noted that these interventions have limited efficacy in maintaining blood glucose levels and place a substantial financial strain on both individuals and the healthcare establishment [2]. Long-term treatments such as pancreatic islet and stem cell transplantation present substantial challenges, rendering these approaches unfeasible for the majority of patients [28]. Findings by various labs have suggested that MSCs-derived exosomes may also have pleotropic roles in DM therapy, slowing the disease’s progression [29]. There are various mechanisms by which MSCs-derived exosomes as a mediators interact with target cells and organs in diabetes (Table 1 and Fig. 1).

**Table 1:** The function of MSCs-derived exosomes in diabetes mellites.

DM type	Exosomes sources	Exosomes reactive molecules	Target cells/Target tissues	Mechanism of action	Effect	Ref
Type 1 diabetes (T1DM)	ADMSCs	unspecified	Splenic mononuclear cells	Decrease inflammatory factors, increase Treg cells ratio	Ameliorate autoimmunity and inflammatory reaction	[34]
	MSCs	unspecified	Th1 and Th17 cells	Downregulate Th1 and Th17 cells differentiation, impede T cells activation, enhance IL-10 levels	Inhibit islet inflammation, and alleviates the disease progression	[7]
	iMSCs	Innate stimuli and unique antigens (unspecified)	Autoreactive T cells and B cells	Activate the autoreactive T and B cells by binding to toll-like receptor, induce IFN-γ expression	Trigger autoimmune responses	[37]
	MenSCs	unspecified	Islets β‐cells	Increase the quantity of islets, the β‐cells mass and insulin production	Induce the islet regeneration	[23]
	BMSCs	unspecified	Islets cells	Stimulate islet cells regeneration, enhance the quantity and size of Langerhans islets, and reduce inflammation and fibrosis	Alleviates the disease progression	[38]
	BMSCs	miRNA-375	Islets	Reduce immune activity, relieve islet damage, and impede apoptosis of transplanted islets	Improve islet transplantation	[41]
	Human MSCs	VEGF	Islets	Preserve islet survival and insulin function by PI3K signaling pathway	Ameliorate islet transplantation outcomes	[8]
Type 2 diabetes (T2DM)	hUCMSCs	unspecified	Muscle, liver, and pancreas	Reverse insulin resistance, relieve β-cell apoptosis and destruction	Alleviates the disease progression	[43]
	hUCMSCs	unspecified	Pancreas, kidney, and liver	Restore islet structure, reduce the HOMA-IR, and enhance insulin sensitivity by GLUT1–4	Ameliorate insulin resistance	[45]
	hUCMSCs	miRNA-21	Islets β‐cells	Protect β-cells against apoptosis by mitigating ER stress and suppressing the phosphorylation of p38 MAPK	Improve islet survival	[47]
	BMSCs	miRNA-29b-3p	Adipocytes, myocytes, and hepatocytes	Enhance insulin sensitivity	Modulate aging-related insulin resistance	[20]
	hUCMSCs	unspecified	Islets	Attenuate insulin deficiency, stimulate pancreatic islets’ regenerative capability via the Extl3-Reg-cyclinD1 pathway	Improve diabetes outcomes	[22]

### MSCs derived exosomes and T1DM

T1DM, also known as autoimmune diabetes, arises from the autoimmune-mediated β-cells apoptosis and dysfunction, resulting in the patients depend on lifelong therapy involving exogenous insulin injections [30]. T1DM arises as a consequence of a complex dialog between immune cells and T cells inside the islet microenvironment. These interactions lead to the production of chemokines and cytokines, which subsequently facilitate the transmission of cell–cell proapoptotic signals [31]. Cytokines activate CD8^+^ cytotoxic–T cells, which in turn recruit and activate immune cells to the islets, resulting in the destruction of β-cells [31, 32]. This dialog is determined by a confluence of environmental and genetic elements as well as ages [30, 33]. The interaction between immune cells and β-cells can initiate the development of insulitis and the gradual impairment and dysfunction of β-cells, mostly through the process of apoptosis [33].

The immunomodulatory impact of exosomes has been extensively demonstrated. A study suggested that adipose-derived mesenchymal stem cells/mesenchymal stromal cells exosomes (ADMSCs-Exos) may have the potential to reduce the concentrations of interferon (IFN)-γ and interleukin-17 (IL-17), while simultaneously augmenting the ratio of regulatory T cells (Treg) in spleen, as well as transforming growth factor beta (TGF-β), IL-4 and IL-10 in mice with T1DM. The application of exosomes can increase the quantity of islets and also improve hyperglycemia symptoms in T1DM mice, which can be attributed to the amelioration of autoimmunity and inflammatory reaction [34].

MSCs-Exos were shown to inhibit development of T helper 1 and 17 cells (Th1, Th17) effectively, leading to the restoration of equilibrium between Th1 and Th2 immune reactions in established mouse models of T1DM [7, 35]. Consistent with these findings, recent research further validated that MSCs-Exos possess the capability to attenuate the Th1 and Th17 cell differentiation, impede the activation of T cells, and enhance the production of IL-10, resulting in the repression of islet inflammation, a substantial increase of plasma insulin, and delaying the onset of T1DM effectively [7]. The islets of Langerhans consist of diverse progenitor cells, which encompass precursor cells for insulin-producing β-cells, and endothelial progenitor cells.

Non-obese diabetic (NOD) mice, which displayed the clinical symptoms similar to those observed in humans with T1DM, including hyperglycemia, polyuria, and polydipsia, are the preferred spontaneous disease model for T1DM [36]. According to in vitro research, Rahman MJ et. al found that islet MSC-like cells (iMSCs) consistently express MSC surface markers, and these iMSCs released exosomes (iMSC-Exos) contain distinct unique antigens and are highly immunostimulatory. It has been observed that these iMSC-Exos could induce early inflammation, stimulate autoreactive B and T cells by binding to toll-like receptors in NOD mice [37]. Additionally, iMSC-Exos also have the ability to elicit comparable levels of IFN-γ production and B-cells proliferation. Therefore, iMSC-Exos might serve as the carrier of autoantigens and perhaps act as the trigger of autoimmune in the NOD mice [37].

Streptozotocin (STZ) was utilized to produce islet destruction and diabetes in animal experiments. Exosomes produced from menstrual blood‐derived mesenchymal stem cells (referred as MenSCs-Exos) were administered intravenously to mice at various time intervals following STZ injection (including 0, 2, or 10 days) and in either single or repeated therapeutic dose. The finding represented that MenSCs-Exos may have the potential to enhance the quantity of islets, improve the mass of β-cells, as well as stimulate synthesis of insulin by pancreatic and duodenal homeobox 1 signaling pathways in diabetic animals after a period of ~6 weeks in cases where β-cells were compromised.

Although exosome therapy resulted in the increased production of insulin, there was no statistical variation detected in non‐fasting blood glucose. This phenomenon may be attributed to several factors, such as the insufficient β-cells regeneration, the immaturity of regenerated islets, or the detection of insulin might be proinsulin form rather than its active form [23]. Additionally, MSCs-Exos have demonstrated superior performance in the regenerative properties and therapeutic efffacy compared to MSCs alone in STZ induced diabetic rats. Within 4 weeks of administration of exosomes released from bone marrow mesenchymal stem cells (BMSCs-Exos), diabetic rats exhibited a notable decrease levels in blood glucose, accompanied by an elevation of plasma insulin. Moreover, histopathological and immune-histochemical examination also revealed islet cells regeneration, accompanied by a rise in the quantity and dimensions of Langerhans islets, and reduction of inflammation and fibrosis. Yet, the aforementioned indices exhibited greater improvement when the rats were subjected to treatment with BMSCs as opposed to BMSC-Exos [38].

Replacing β-cells by the transplantion of pancreatic islets has demonstrated relatively safe and efficacy in the treatment of T1DM, would provide T1DM patients a “functional cure” [39, 40]. Despite recent advancements in therapy, the extensive utilization of islet transplantation remains constrained due to the immunological rejection and graft failure. The activation of proinflammatory cytokines induces the upregulation of apoptosis and hypoxia-associated proteins or miRNAs, such as Caspase-3, iNOS, Fas, and miR-375, causing the dysfunction of newly implanted islets. Notably, BMSCs-Exos transfected with pshFas-anti-miR-375 could result in the downregulation of Fas and miR-375 levels, reduce immune activity, relieve islet damage against inflammatory cytokines and inhibit early apoptosis of transplanted islets [41]. Furthermore, MSCs have been used for the purpose of safeguarding isolated islets through the production of vascular endothelial growth factor (VEGF), which act as an anti-apoptotic and pro-survival agent, preserving the integrity of the islets and contributes to the process of revascularization of transplanted islets [42]. A recent study revealed that MSC-Exos, as the main paracrine therapeutic mediators of MSCs, have the ability to preserve islet survival and enhance the production of insulin in vitro through the activation of pro-survival phosphoinositide 3-kinase (PI3K) pathways [8]. In quite recent future, exosome therapy, safe, effective, a cell-free therapeutic approach, has the potential to significantly enhance islet function and ultimately enable patients to decrease or even eliminate insulin usage.

### MSCs derived exosomes and T2DM

T2DM is the predominant phenotype of diabetes, exceeding 90% of the diabetic population [1, 2]. T2DM is primarily attributed to insulin resistance coexists with insulin relative deficit owing to β-cells dysfunction. Glucose transporter 4 (GLUT4), which serves as the primary glucose transporter, is notably observed in adipose tissue and skeletal muscle. A recent study established a T2DM rat model of by the utilization of a high-fat diet (HFD) and streptozotocin. They claimed that exosomes released from human umbilical cord mesenchymal stem cells (hUCMSCs-Exos) could not only indirectly reverse insulin resistance to enhance glucose metabolism, increase glycogen storage in liver, but also suppress β-cells apoptosis induced by STZ, consequently restoring the insulin-secreting function and sustaining glucose homeostasis in rat with T2DM. Specifically, hUCMSCs-Exos restored the tyrosine kinase phosphorylation site of protein kinase B and insulin receptor substrate1 (IRS-1) in the diabetic rat model, accompanying increased GLUT4 levels and the membrane translocation of GLUT4 in muscle [43]. Compelling evidence suggested that the presence of structural disorder and cumulation of islet amyloid in the Langerhans islets played a crucial role in impairment of β-cells function and development of T2DM [44]. Additionally, it also has been documented that the utilization of HucMSC-Exos has the potential to reinstate the structural integrity of islets, augment insulin sensitivity by the facilitation of glucose uptake through GLUT1–4, as well as diminish the insulin resistance in rats with T2DM [45].

The potential impact of hypoxic stress on the deterioration of β-cells function is worth considering. Under situations of hyperglycemia, β-cells experience hypoxia as a consequence of heightened oxygen consumption. This hypoxic state triggers a specific downregulation of various genes, namely *Wfs1*, *Mafa*, *Pdx1*, *Ndufa5*, *Slc2a2*, *Ins1*, *Kcnj11*, *Foxa2*, and *Neurod1*, which eventually leads to apoptosis and dysfunction of β-cells in diabetic mice [46].

A study indicated that HucMSC-Exos had the ability to protect β-cells against apoptosis in hypoxic conditions by alleviating endoplasmic reticulum (ER) stress generated by hypoxia and suppressing apoptotic signal pathways, reducing gluconeogenesis, while simultaneously enhancing hepatic glycolysis. Additionally, hUCMSCs-Exos was also shown to suppress p38/mitogen-activated protein kinase (P38/MAPK) pathways of β-cells, which is facilitated by highly abundance of let-7g and miRNA-21 [47]. In conclusion, these findings suggest that hUCMSCs-Exos could mitigate the loss of β-cells, improve insulin resistance, and maintain glucose homeostasis on T2DM rats.

A strong association exists between increasing age and T2DM, such that its incidence is much greater in the older individuals compared to younger individuals [48]. The elderly often develop insulin resistance in T2DM. Recent findings have revealed that administration of aged mice BMSCs-derived exosomes had a notable impact on the insulin-stimulated glucose uptake, both in vivo and in vitro. Furthermore, it has been observed that this intervention leads to a reduction in insulin sensitivity, as evidenced by elevated fasting blood glucose, serum insulin levels, and the homeostatic model assessment of insulin resistance index (HOMA-IR) in young wild-type mice. Then, they further found an elevation of miRNA-29b-3p in BMSCs-Exos during ageing. Sirtuin 1 (SIRT1) serves as the immediate downstream mediator miRNA-29b-3p in negative modulation of insulin sensitivity. Collectively, these findings indicated that BMSCs-Exos miR-29b-3p has the ability to regulate insulin resistance associated with ageing [20]. This discovery highlights the possibility of targeting miR-29b-3p as a therapeutic approach for managing insulin resistance in the context of aging.

Promoting islets regeneration and augmenting insulin secretion are predominant goals to restore T2DM. The hUCMSCs-Exos enhanced proliferation of cells within Langerhans islets and insulin production, reduced blood glucose level, mitigated damage to pancreatic tissue, and ultimately improved disease outcomes through the regulation of Extl3-Reg-cyclinD1 pathway in STZ-induced diabetic mice [22]. Oh et al. utilized numerous exosomes that were produced from murine pancreatic β-cells and designed a method for the effeciently differentiating insulin-producing cells [49]. They effectively demonstrated that bone marrow stem cells were induced successfully by exosomes into insulin-producing cells in diabetic experimental animals, resulting in a subsequent reduction levels of blood glucose.

In summary, MSCs-Exos may stimulate intrinsic regenerative capabilities of pancreatic islets, alleviate insulin resistance, and restore the β-cells function. MSCs-derived exosomes display a potential capacity for the management of T2DM, whereas further additional investigation is necessary before their clinical application can be considered.

## MSCs derived exosomes and diabetic complications

DM is frequently accompanied by severe and possibly life-threatening complications, which are mostly caused by elevated blood glucose induced cellular or molecular dysfunctions of microvascular and macrovascular [3]. Complications that have been strongly associated with DM include both macrovascular and microvascular complications, including DKD, retinopathy and peripheral neuropathy (microvascular), and peripheral arterial disease, ischemic heart disease, and cerebrovascular disease (macrovascular). As MSCs-derived exosomes demonstrated significant immunomodulatory and regenerative potential, a multitude of researches have been undertaked to examine the potential of MSCs-Exos as a viable regenerative intervention and a powerful therapeutic candidate for treating diabetes complications. In the subsequent sections, we would examine advances research discoveries about MSCs-Exos for the mechanisms and applications in various complications of diabetes.

### Diabetic kidney disease

**Fig. 2:**
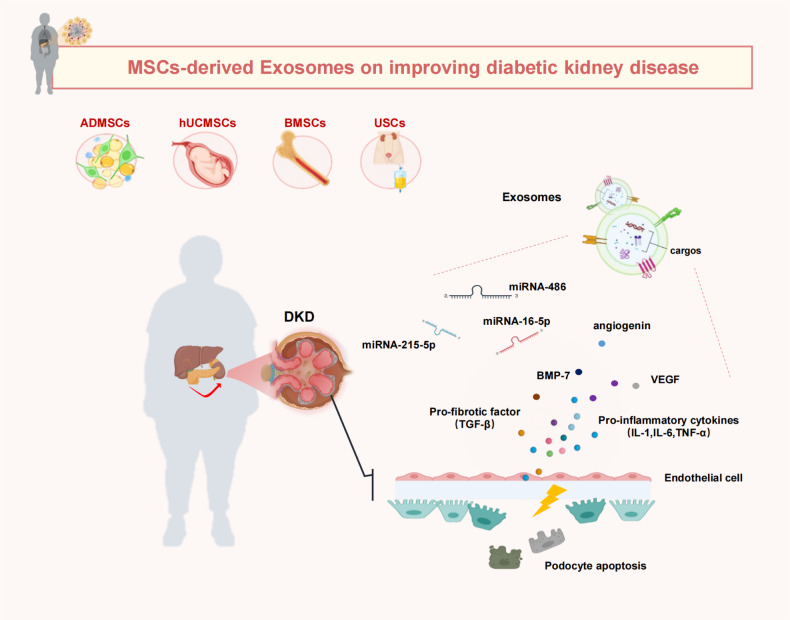
Effect of exosomes derived from MSCs on ameliorating diabetic kidney disease. Exosomes from MSCs containing a variety of miRNAs, cytokines, and growth factors have shown significant efficacy in protecting the function of podocyte and endothelial cells, with a notable preventative impact on the DKD progression.

DKD is a highly detrimental long-term consequence of diabetic microvascular and the major contributor to end-stage renal disease (ESRD) worldwide, primarily distinguished by the consistent presence of proteinuria or a sustained decrease in glomerular filtration rate [50]. Multiple pathophysiological disruptions contribute to the initiation and advancement of DKD, including increased oxidative stress, alterations in hemodynamic, the hypertrophy of glomerular basement membrane, proliferation of mesangial cell, apoptosis and depletion of podocytes, as well as the development of glomerulosclerosis and fibrosis [51]. These multifactorial pathogenic processes implied that a combination of multiple agents will be necessary for the treatment of DKD. The present therapeutic modalities for DKD, such as hemodialysis or transplantation, delay but do not prevent DKD development and have several limitations including high cost and the unpredictable availability of organ for transplantation [28]. Therefore, the development of innovative and effective treatment approaches is required urgently to protect renal function, counteract and reverse the progression of DKD. Exosomes from various tissues, harboring a multitude of growth factors and therapeutic noncoding RNAs, have substantial efficacy in enhancing renal function, and hold promising potential as an innovative therapeutic modality for the management of DKD (Fig. 2 and Table 2).

**Table 2:** Effect of exosomes derived from MSCs on improving diabetic kidney disease.

DM complication	MSC type	Exosomes reactive molecules/Genetic modification/Biomaterials	Mechanism of action	Effect	Ref
Diabetic kidney disease (DKD)	ADMSCs	miRNA-215–5p	Alleviate DKD by inhibiting histone deacetylase 1 and endothelin-1	Alleviate the mesangial hyperplasia and kidney fibrosis	[53, 88]
	hUCMSCs	miRNA‐16‐5p	Suppress VEGFA levels and protects podocytes from the effects of hyperglycemia and apoptosis	Ameliorate DKD through protection of podocyte	[59]
	USCs	VEGF, TGF-β1, BMP-7 and angiogenin	Prevent podocytes apoptosis, and promote glomerular endothelial cell proliferation	Inhibit podocyte apoptosis and promote vascular regeneration	[89]
	BMSCs	unspecified	Suppress abnormal immune cell infiltration, reduce proinflammatory cytokines by downregulating intercellular adhesion molecule 1	Promote renal function recovery	[64]
	ADMSCs	miRNA-215–5p	Inhibit hyperglycemia evoked EMT progression and podocytes migration and apoptosis through suppressing ZEB2	Attenuates epithelial-mesenchymal transition of podocytes	[66]
	BMSCs	unspecified	Autophagy induction and antifibrotic effect	Promote renal function recovery	[72]
	ADMSCs	miRNA-486	Inhibit apoptosis in podocyte	Podocyte repair	[73]
	UCMSCs	unspecified	Inhibit TGF-β1-triggered myofibroblast trans-differentiation and cell proliferation	Reduce kidney inflammation and improve kidney function	[80]
	hUCMSCs	unspecified	Reduce pro-inflammatory and pro-fibrotic cytokines	Reduce kidney inflammation	[79]

#### Alleviate the mesangial hyperplasia

The generation of mesangial matrix by glomerular mesangial cells (GMCs) are important for kidney glomerular homeostasis. Hyperglycemia promotes GMC activation, which commonly results in the increased cellularity of the glomerulus together with expansion of the glomerular stalk and the glomerular matrix. This process eventually leads to the increasing fibrosis and scarring of glomerular mesangium, which are prominent characteristics of DKD referred to as glomerulosclerosis [52]. Notably, a recent work has indicated that exosomes derived from ADMSCs could suppress the excessive proliferation of hyperglycemia-induced GMCs imitating a diabetic nephropathy condition in vitro. In addition, the ADMSCs-Exos also significantly suppressed the production of IL-6 in GMCs, which is a well-known autocrine growth factor that contributes to glomerular damage and the development of mesangial hyperplasia [53]. Further, they extend the findings to an in vivo setting, found that the administration of ADMSCs-Exos effectively mitigated mesangial hyperplasia and kidney fibrosis in DKD rats [53]. Increased concentrations of collagen I and fibronectin have been identified as significant elements in the progression of renal fibrosis and the accompanying deterioration of renal function [54]. ADMSCs-Exos were also found to effectively suppress the increased expression of fibrosis-associated factors in renal tissue of rats with DKD. These effects supported the protective effect of ADMSCs-Exos on mitigating mesangial hyperplasia, renal fibrosis and functional impairment. Importantly, it was observed that miR-125a had a role in the aforementioned protective events facilitated by ADMSCs-Exos in the kidney tissues in DKD rats [53]. Consistent with the above findings, miR-125a, which acts as a direct modulator of IL-6R, have also been already shown by other studies to play an important role in diabetic nephropathy individuals, with a notable preventative impact on the disease progression [55]. Collectively, above mentioned findings collectively indicate that ADMSCs-Exos provide a viable approach to ameliorate mesangial hyperplasia, renal fibrosis as well as enhance kidney function.

#### Podocyte repair

Podocytes, which are specialized epithelial cells located in the glomerulus, are an important part to maintain the charge barrier function together with vascular endothelial cells (ECs) [56]. Research has indicated that hyperglycemia has the potential to induce podocyte apoptosis, resulting in proteinuria, with or without loss of renal function owing to glomerulosclerosis [57]. Hence, the preservation of podocytes against hyperglycemia-induced injury is of utmost importance and has significant therapeutic implications in the management of DKD.

### Regulation of VEGF expression

The significance of VEGF in the pathophysiology of DKD has shown contradictory conclusions. Several studies have indicated that overexpression of VEGF increases the vascular ECs’ permeability, proliferation and migration, as well as the induction of matrix-degrading protease, leading to the increased thickeness of glomerular basement membrane, resulting in eventually progression of DKD [58]. Therefore, the inhibition of VEGF overexpression may serve as a prospective approach in mitigating the progression of DKD. The researchers indicated that upregulation of miRNA‐16‐5p in hUCMSCs-Exos suppress VEGFA levels and provides protection to podocytes against the detrimental effects of apoptosis and hyperglycemia, both in vitro and in vivo [59]. Then they injected exosomes harboring an elevated expression of miRNA-16–5p into diabetic rats through caudal vein. The results indicated that urine-derived stem cells (USCs), overexpressed miRNA-16–5p, exhibited protective properties on podocytes in the diabetic rats [59]. Down-regulated miR-26a-5p were identified in diabetic nephropathy, thereby could act as diabetic nephropathy biomarkers and therapeutic mediators [60]. Elevated expression of miRNA-26a-5p in ADMSCs-Exos also resulted in the suppression of glomerular podocytes apoptosis, primarily by the downregulation of VEGFA [61].

Intriguingly, VEGF’s low level may have detrimental effects by diminishing the number of podocytes and exacerbating the development of renal disease [58], enhancing the expression of VEGF might serve as a approach to improve DKD. In another study, USCs-Exos, contained various potential factors, including angiogenin, TGFβ1, and bone morphogenetic protein-7, could potentially decrease the production of caspase-3, prevent podocytes apoptosis, and enhance the expansion of glomerular ECs in diabetic rats [62]. Considering these contradictory findings, more research would be needed to comfirm the comparative efficacy of different strategies for regulating VEGF in DKD treatment.

### Suppression of inflammation

The etiology of DKD is intricate and encompasses a variety of interconnected mechanisms. Infiltration of immune cells, particularly macrophages, is commonly detected in kidney tissues at all stages of DKD. Elevated levels of inflammatory cytokines, chemokines, adhesion molecules and growth factors, such as tumor necrosis factor (TNF) IL-6, TGFβ, intercellular adhesion molecule 1 (ICAM-1), and VEGF, has also been documented in patients with DKD [63]. Hence, the inhibition of invasion by inflammatory cells and the generation of proinflammatory cytokines may function as a defensive mechanism for the kidneys against deleterious development of DKD. The use of BMSCs-Exos has been found to have a significant role in meliorating DKD, through inhibiting excessive infiltration of immune cells and mitigating the generation of proinflammatory factors by downregulating the ICAM-1 expression [64]. In another investigation, elevated miRNA‐16‐5p in human USCs-Exos have the ability to inhibit the expression of inflammatory cytokines TNF‐α and chemokines CCL2 (also known as MCP-1) in the nephrocytes of rats with diabetes, resulting the downregulation of VEGFA expression, prevention of podocytes apoptosis, eventually with accompanying promotion of podocyte proliferation [59].

### Epithelial-to-Mesenchymal Transition (EMT) suppression

The hyperglycemia-indeced podocytes injury may be identified by the process of EMT and migration resulting in podocytes loss, which is recognized as a significant causative factor of the glomerular filtration barrier destruction and proteinuria fromation, ultimately contributing to DKD development [65]. Hence, inhibition of the EMT has promised as a viable strategy to mitigate DKD progression. Notably, ADMSCs-exosomes, transporting miRNA-215–5p to podocytes, were found to inhibit hyperglycemia-occasioned EMT development, and the podocytes migration and apoptosis. Mechanically, miRNA-215–5p was found to regulate such effect by suppressing zinc finger E-box-binding homeobox-2 (ZEB2) expression [66]. ZEBs are transcription factors that bind to DNA and have extensively documented functions in initiating EMT and facilitating cell migration [66]. Mechanistically, ZEB2 has the ability to engage in a mechanistic interaction with Sp1 (a transcription factor), therefore facilitating the activation of mesenchymal genes, such as integrin α5, consequently leading to the enhancement of EMT process and facilitation of cell migration in human cancer cells [67]. Besides, ZEB2 also has the ability to directly interact with conserved E2 boxes located on the promoter region of E-cadherin, leading to the repression of E-cadherin production and subsequently promoting cellular invasion, thereby accelerating the process of EMT [68].

### Autophagy induction

Autophagy is a meticulously controlled homeostatic process that serves to eliminate cytotoxic protein aggregates and impaired organelles by lysosomal degradation [69]. The activation of mTOR was crucial to the preservation of glomerular podocyte function, as well as the occurrence of glomerular and tubular cell hypertrophy and podocytes damage, on the contrary, mTOR inhibition might preserve podocytes and prevent the progression of diabetic nephropathy [70]. Impairment of autophagy leads to the cumulation of collagen in extracellular matrix, dysfunction of mitochondria in podocytes, and elevated levels of ER stress in tubular cells [71]. BMSCs-derived exosomes could ameliorate renal function as well as promote repairation of renal tissues by the regulation of autophagy via inhibiting the mTOR signaling pathway, suppressing TGF-β expression, and reducing fibrosis in diabetic rats [72]. Similarly, ADMSCs-Exos containing an elevated concentration of miR-486 have been shown to vividly reduce podocyte injury and ameliorate DKD symptom, through inhibiting Smad1/mTOR signaling pathway in podocyte [73]. These findings indicated that MSCs-Exos may have the capacity to mitigate kidney damage caused by diabetes through the facilitation of vascular regeneration and cell survival. MSCs-Exos might be a promising therapeutic intervention for DKD.

#### Enhancement of renal fibrosis

Fibrosis is a pivotal part in DKD, eventually resulting in permanent kidney injury. Multiple studies have demonstrated that TGF-*β*1 can interfere with the cell cycle, stimulate the production of extracellular matrix, therefore expediting the process of renal fibrosis [74]. TGF-*β*1 triggers the trans-differentiation of the intrinsic cells not only via the downstream Smad2/3-dependent pathways, but also stimulates the Smad2/3-independent signaling, including MAPKs, Wnt/*β*-catenin, RhoA, and PI3K/Akt, which are implicated in renal fibrosis [75–78]. The administration of hUCMSCs-Exos has been shown to have a positive impact on renal inflammation and fibrosis by decrease of pro-inflammatory cytokines (IL-1β, IL-6, TNF-α) as well as pro-fibrotic factor (TGF-β) in both kidney and bloodstream of DKD rats. Additionally, hUCMSCs-Exos also exhibited significant secretion of several growth factors, including fibroblast growth factor, epidermal growth factor, hepatocyte growth factor, and VEGF [79]. Consistent with this, another investigation has documented that exosomes obtained from umbilical cord MSCs can decrease the accumulation of collagen I and fibronectin through inhibition of myofibroblast trans-differentiation and cell proliferation, which is triggered by TGF-*β*1. These inhibitory effects are mediated through the MAPK and PI3K/Akt pathways. Additionally, expression of MMP2 and MMP9 are elevated as a result of exosome treatment [80]. As previous mentioned, MSCs-Exos have been found to suppress the release of TGF-β1 to reduce EMT and interdict the mesangial cells proliferation, thereby alleviating renal fibrosis [72].

### Diabetic wounds healing

**Fig. 3:**
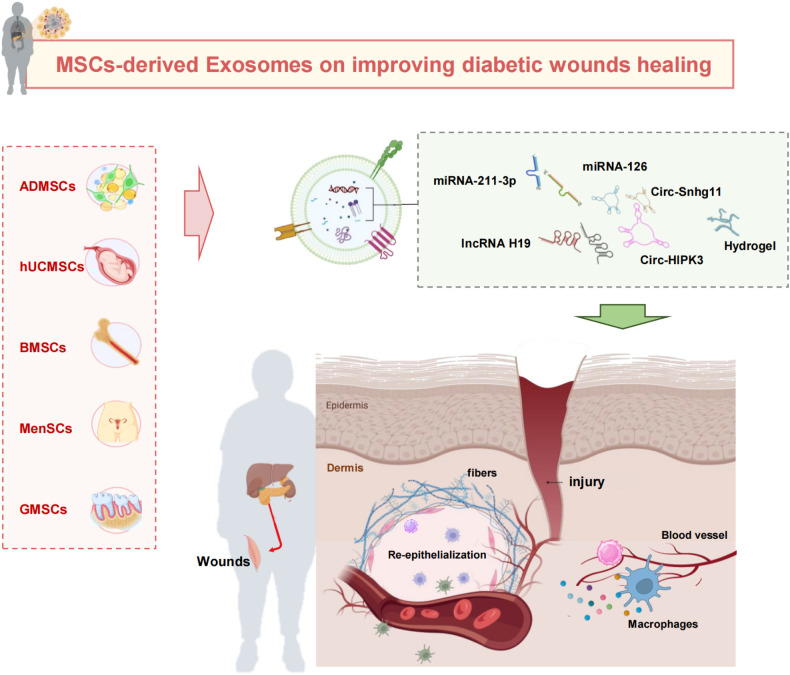
Impact of exosomes derived from MSCs on the enhancement of healing in diabetic wounds. This figure depicts that MSCs-derived exosomes have the potential to promote wound healing through mechanisms that modulate macrophage polarization, promote endothelial cells angiogenesis, accelerate the process of re-epithelialization, and enhance collagen deposition.

Diabetic wounds have delayed healing as a result of raised generation of reactive oxygen species (ROS), chronic inflammation, impaired angiogenesis, and delayed re-epithelialization [81]. Diabetics foot ulcers remained the primary reason of non-traumatic amputation, despite advancements in wound care, which lead to poor life quality and great burden on patients and the healthcare system. The conventional clinical management of diabetic wounds, which involves procedures such as surgical debridement and the application of negative pressure treatment with wound dressings, appears to be ineffectual for a significant number of patients [82]. This ineffectiveness can be attributed to the reduced angiogenic and cellular function observed in the vicinity of the wound sites. Therefore, Thus, the use of techniques aimed at enhancing angiogenic responses and cellular activity at the sites of wounds might potentially provide benefits in expediting the healing process of diabetic wounds. As shown in Table 3 and Fig. 3, MSCs-exos has the ability to facilitate the process of wound healing through various mechanisms [11].

**Table 3:** Impact of exosomes derived from MSCs on the enhancement of diabetic wounds healing.

DM complication	MSC type	Exosomes reactive molecules/ Genetic modification/Biomaterials	Mechanism of action	Effect	Ref
Diabetic wounds healing	ADMSCs	circ-Snhg11	Suppress endothelial cell injury and induce macrophages polarization through miRNA-144–3p/HIF-1α pathway	Vascularization and anti-inflammation	[86]
	BMSCs	unspecified	Increase the polarization ratio of M2 to M1 macrophages via PTEN/AKT pathway	Suppress inflammatory response	[85]
	BMSCs	lncRNA H19	Regulate inflammation and apoptosis of fibroblasts by inhibiting miRNA-152–3p-induced PTEN suppression through PI3K/AKT pathway	Regulate inflammation	[87]
	ADMSCs	Overexpressed Nrf2	Reduce ROS, NOX1, NOX4 and inflammatory cytokines	Vascularization and anti-inflammation	[90]
	hUCMSCs	unspecified	Reduce oxidative stress, improve cell proliferation	Promote angiogenesis and suppress inflammatory response	[91]
	ADMSCs	unspecified	Enhance the migration of vascular endothelial cells, alleviate mitochondrial function, inhibit inflammatory reactions by modulating SIRT3/SOD	Vascularization and anti-inflammation	[91]
	BMSCs	unspecified	Enhance angiogenesis and induce viability of fibroblasts by ERK 1/2, Akt, and STAT3 pathways	Vascularization	[94]
	MenSCs	unspecified	Induce M1-M2 polarization, enhance neoangiogenesis via VEGFA	Vascularization, re-epithelialization and anti-inflammation	[95]
	BMSCs	miRNA-211–3p	Augment the angiogenesis via AKT/eNOS pathway	Enhance angiogenesis	[96]
	BMSCs	miRNA-126	Stimulate angiogenesis via miRNA-126 mediated PTEN protein downregulation	Vascularization	[97]
	hUCMSCs	unspecified	Regulate oxidative stress injuries of endothelial cells	Vascularization	[91]
	hUCMSCs	circ-HIPK3	Promote angiogenesis by decreasing miR-20b-5p and upregulating Nrf2 and VEGFA	Vascularization	[99]
	hUCMSCs	unspecified	Stimulate β-catenin activation inside endothelial cells	Vascularization	[100]
	hUCMSCs	unspecified	Promote angiogenesis via transferring DMBT1 protein	Vascularization	[101]
	ADMSCs	eHSP90	Reduce hyperglycemia-induced endothelial cell damage	Vascularization	[103]
	ADMSCs	mmu_circ_0000250	Enhance wound vascularization by the autophagy by miR-128–3p/SIRT1	Vascularization	[105]
	Human ADMSCs	miRNA-21–5p	Increase re-epithelialization, vessel maturation and collagen remodeling by Wnt/b-catenin pathway	Enhance vascularization and re-epithelialization	[106]
	Synovium MSCs	miRNA-126–3p	Accelerate re-epithelialization, promote collagen maturity throughMAPK/ERK, PI3K/AKT pathways	Enhance angiogenesis, accelerate re-epithelialization	[107]
	HypoxicADMSCs	miRNA-21–3p, miR-31–5p, miR-126–5p (upregulated) and miR-99b, miR-146a (downregulated)	Promote fibroblasts proliferation and migration by PI3K/Akt pathway, enhance extracellular matrix production	Suppress inflammation responses	[111]
	ADMSCs	unspecified	Stimulate re-epithelialization, contraction, and angiogenesis by TGF-β/Smad3 pathway	Accelerate re-epithelialization and enhance angiogenesis	[112]
	GMSCs	Chitosan/silk hydrogel sponge	Remodel of collagen, enhance angiogenesis and neuronal growth	Vascularization and re-epithelialization	[113]
	ADMSCs	Hydrogel	Promote angiogenic potential of endothelial cells	Vascularization and re-epithelialization	[114]

#### Anti-inflammation

Individuals with diabetes have a persistent state of long-term inflammation, characterized by the presence of pro-inflammatory macrophages in injured site which exhibit limited ability to change into the anti-inflammatory M2 macrophages [83]. In diabetic wounds, an overabundance of polarization in M1 macrophages leads to an inevitable escalation of proinflammatory cytokines, including TNF‐α and IL-1β, which in turn prolongs the inflammatory period of wound repair [84].

MSCs-exosomes have demonstrated efficacy in mitigating inflammation through inducing the polarization of M1-M2 macrophages, which process contributes to the reduction of inflammation in injury sites and facilitates the healing process [85]. Under hyperglycemia conditions, circ-Snhg11 expression decreased. Through the participation of the miR-144–3p/HIF-1 axis, circ-Snhg11 overexpression exosome from ADMSCs may suppress hyperglycemia-induced endothelial cell damage and drive the polarization of macrophages towards M2 phenotype [86]. MSCs-derived exosomes exert effects on the healing process through similar pathways, with the PTEN/PI3K/AKT pathway being extensively investigated and recognized as particularly significant. Recent research has demonstrated that BMSCs-Exos could enhance the process of wound repairing by inhibiting inflammatory reaction, which was achieved by modulating the balance between M1 and M2 macrophages through the activation of the PTEN/AKT pathway [85]. Similarly, Li and colleagues discovered that BMSCs-Exos, contained lncRNA H19, exhibited a protective impact against inflammation and apoptosis in fibroblasts by disrupting the inhibition of PTEN through miR-152–3p, stimulating healing in a diabetic foot ulcers rat model [87]. The close association between the PETN/PI3K/AKT axis and macrophage polarization was evident, as it played a pivotal role in regulating localized inflammatory reactions at the injury site. This regulation facilitates the swift transition from the inflammatory stage to tissue regeneration phase, thereby enhancing the healing process of diabetic wounds.

ADMSCs-Exos have demonstrated in a growing number of studies to be an essential intercellular communication medium that can mitigate inflammatory reaction and have a promising potential in wound healing. Hyperglycemia accelerates the premature senescence of ECs, while also increases the generation of ROS and inflammatory factors in wounds [88]. Nuclear factor-E2 related factor 2 (a transcription factor, Nrf2) is involved in several cellular processes, such as cell migration, proliferation, differentiation, and apoptosis. Additionally, it serves to protect against oxidative stress and regulates the functioning of antioxidant enzyme through the mediation of antioxidant response elements (ARE) [89]. Xue Li et al. assessed physiological traits in diabetes patients and found considerably higher levels of fasting glucose, glycated hemoglobin, as well as inflammatory markers (NOX1, NOX4) [90]. They also examined the therapeutic effects of ADMSCs-exos and have shown that high Nrf2 expression in ADMSCs-exos could reduce ROS and inflammatory cytokines [90]. The comparable findings were also verified in hUCMSCs. A study demonstrated that hUCMSCs-Exos providing a promising approach for accelerating the repairing of diabetic cutaneous wounds by stimulating cell proliferation and reducing the content of inflammatory factors and oxidative stress in human umbilical vein endothelial cells (HUVECs) under high glucose environment [91]. SOD is an enzyme that functions as scavenger of superoxide anion free radicals, therefore contributing significantly to the preservation of cellular redox equilibrium [92]. Zhang et al. discovered that ADMSCs-Exos has a ability to reduce the accumulation of ROS and inflammatory reactions by modulating SIRT3/SOD2, thereby speeding up the healing of diabetic wounds [93].

#### Promoting angiogenesis

The degree of wound vascularization is a critical factor that significantly impacts the rate of healing and the subsequent process of remodeling. Nevertheless, hyperglycemia is associated with a decline in the concentration of pro-angiogenic factors, impairment of endothelial function, and reduction of blood vessel development; consequently, angiogenesis is frequently blocked in diabetes patients, resulting in delayed wound healing [82]. MSCs-Exos have garnered attention as potential therapeutic candidates for addressing delayed wound repairment in individuals with diabetes, owing to an ability to enhance the angiogenesis.

In a recent study, the effects of ADMSCs-Exos on the wound healing process was examined using a full-layer back skin damage model with diabetes. The findings of the study revealed that ADMSC-exos led to an upregulation in the levels of angiogenesis-associated proteins, specifically VEGF, Fetal Liver Kinase-1 (FILK1), and Angiopoietin-1 (ANG1), but a downregulation of endogenous angiogenesis inhibitors, namely Thrombospondin-1 (TSP1) and Vasohibin 1 (VASH1). Consequently, the migration and activity of vascular ECs are raised, thereby leading an increased potential for angiogenesis [93]. In addition, ADMSC-exos with highly expressed Nrf2 could enhance the proliferation of ECs and stimulate angiogenesis via enhancing the phosphorylation of SMP30, VEGF, and VEGFR2 [90]. The study revealed that BMSCs-Exos possess the capacity to initiate various pathways (ERK 1/2, Akt, and STAT3), which are crucial in the process of healing and stimulate the production of several trophic factors [stromal-derived growth factor-1 (SDF1), insulin-like growth factor-1 (IGF1), hepatocyte growth factor (HGF), and nerve growth factor (NGF)] [94]. It was also observed that menstrual blood mesenchymal stem cell exosomes promoted the formation of new blood vessels through VEGFA upregulation [95]. Yu et al. illustrated that the administration of BMSCs-Exos can augment the process of angiogenesis in ECs via the upregulation of miRNA-211–3p and activation of AKT/eNOS (endothelial nitric oxide synthase) signaling, thereby resulting in an expedited wound vascularization and promoting the regeneration of diabetic rats [96]. Additionally, the finding conducted by Ding et al. provided an evidence that exosomes originated from BMSCs preconditioned by deferoxamine could activate PI3K/AKT pathway via the downregulation of PTEN mediated by miR-126, leading to the stimulation of angiogenesis in vitro, and the enhancement of wound repairment in vivo [97].

Emerging studies have indicated that hUCMSCs-Exos can stimulate angiogenesis and augment tissue regeneration [98]. Notably, hUCMSCs-Exos significantly suppressed oxidative stress induced by hyperglycemia, promoting cell proliferation, cellular activity, and angiogenesis of HUVECs [91]. Liang ZH et al. demonstrated that circHIPK3 in hUCMSCs-Exos enhanced cell proliferation, migration, and angiogenesis through the downregulation of miRNA-20–5p, resulting in the upregulation of Nrf2 and VEGFA mechanistically. These results demonstrated that UCMSCs-derived exosomes circHIPK3 might be a therapeutic approach for the management of diabetic ulcers, through providing protection to high glucose-treated HUVECs by the regulation of miR-20b-5p/Nrf2/VEGFA axis [99]. Moreover, Zhang et al. illustrated that hUCMSCs-Exos facilitate the activation of β-catenin of ECs by the action of Wnt4. This activation subsequently leaded to the dose-dependent enhancements on proliferation and migration, ultimately resulting in proangiogenic effects [100]. Exosomes from human USCs exhibited an enrichment of proteins, particularly the pro-angiogenic factor known as deleted in malignant brain tumors 1 (DMBT1), which are involved in governing the biological processes associated with wound healing. Human USCs derived exosomes could promote angiogenic responses cascade in ECs, improve angiogenesis, and facilitate wound repairment through DMBT1 in mice with diabetes [101]. These results suggested that exosomes from human USCs may hold potential as a viable strategy for the management of soft tissue wound repair in individuals with diabetes.

The involvement of oxidative damage is of notable importance in the pathogenesis of diabetic wounds. The presence of a hypoxic environment has been found to have advantageous impacts on the survival and genetic integrity of MSCs [102]. Hypoxia preconditioned ADMSCs-exos could reduce hyperglycemia-induced ECs damage, increase the differentiation of endothelial progenitor cell, and ultimately enhance healing in diabetic mice [86]. Sen et al. also found that ADMSCs-Exos had the ability to release eHSP90, which mitigated cellular damage induced by hypoxia and oxidative stress, as well as promoted migration, proliferation, and angiogenesis in fibroblasts, keratinocytes, and ECs. Eventually, the administration of ADMSCs-Exos directly at the sites of wounds resulted in a significant enhancement in collagen deposition and neovascularization [103]. Consistent with this, a recent study shown that hyperglycemia contributes to the early senescence of ECs, and excessive levels of Nrf2 amplifies the impact of ADMSCs-Exos in mitigating the senescence of ECs caused by high glucose levels and improving vascularization in STZ-induced diabetic rat model, possibly by inhibiting ROS [90].

Recently, it has been found that MSCs-Exos have been shown a significant contribute to the process of vascularization in skin wounds and the progression of wound healing [104]. Researchers discovered that mmu_circ_0000250 exhibited therapeutic properties on vascular ECs that had been subjected to high glucose circumstances. Moreover, the exosomes derived from ADMSCs treated with mmu_circ_0000250 could enhance the upregulation of SIRT1 by adsorbing miRNA-128–3p, encourage vascularization, and eventually expedite the repairment process of DM wounds [105]. In addition, it was also shown that ADMSCs-Exos containing a significant amount of mmu_circ_0001100 (circ-Snhg11) played a considerable role in enhancing diabetic wound repair by stimulating miRNA-144–3p/HIF-1a/VEGF pathway [86]. Furthermore, MSCs-Exos can be used as a viable vehicle for delivering therapeutic noncoding RNA, hence facilitating the diabetic wounds healing process. Qijun Lv et al. devised a human adipose stem cells-derived exosomes (hADMSCs-Exos)-based miRNA delivery approach to boost its curative effectiveness and found that the combination of miRNA-21–5p and ADMSCs-Exos resulted in an upregulation of MMP-7 by stimulating the Wnt/β-catenin pathway. The modified MSCs-Exos containing miR-21–5 exhibited significant efficacy on enhancing the migration and proliferation of keratinocytes, and expediting the process of mending diabetic wounds by promoting of collagen remodeling, re-epithelialization, and vessel maturation [106]. Additionally, a recent study evaluated a potential therapeutic approach involving controlled release of synovium mesenchymal stem cells (SMSCs) derived exosomes that overexpress miRNA-126–3p in conjunction with chitosan. This approach involved overexpressing miRNA-126–3p and transferring the enhanced angiogenic capacity from endothelial progenitor cells to SMSCs. Subsequently, it was discovered that the composite material could release exosomes in a sustainable manner and significantly stimulate the MAPK/ERK and PI3K/AKT pathways, all of which were crucial for activating angiogenesis, accelerating re-epithelialization, promoting of collagen maturation in diabetic wounds and enhancing overall efficiency of the healing process [107]. These findings suggested that MSCs-Exos, which are cell-free particles, hold significant potential as a promising candidate for pro-angiogenic treatment in diabetic wounds.

#### Accelerating re-epithelialization and collagen deposition

Re-epithelialization, a crucial process that modulates of chronic wound healing through keratinocytes cell migration, aims to restore the epidermal barrier. The presence of a hostile microenvironment in diabetic wounds might obstruct the migration of epidermal cell and the process of re-epithelialization, thereby limiting the chronic wound healing process [108]. ADMSCs-Exos has the potential to expedite the process of re-epithelialization, enhance collagen deposition, and promote wound healing.

The excessive ROS resulted in the continuous generation of pro-inflammatory factors and excessive synthesis of matrix metalloproteinase, especially in turn decreased the granulation tissue formation, hampered neovascularization, and impaired extracellular matrix deposition, ultimately resulting in delayed wounds repairs [109, 110]. The study revealed that hypoxic adipose stem cell exosomes (HypADMSCs-exo) have a role in adapting to hypoxic conditions and facilitating the repair process of diabetic wounds. Compared to ADMSCs-Exos, the expression levels of 215 microRNAs were found to be increased, whereas 369 miRNAs were shown to be decreased in HypADMSCs-Exos. Several microRNAs, including miRNA-21–3p, miRNA-31–5p, miRNA-126–5p (up-regulated), and miRNA-99b, miRNA-146a (down-regulated), have been identified as significant contributors to the migration and proliferation of fibroblasts, and TGF-β activity, via activating the PI3K/AKT signaling pathways associated with immune response [111]. The HSP family is comprised of a collection of extensively conserved proteins that exhibit a response to many stressors, including but not limited to heat, hypoxia, trauma, and hunger. ADMSCs-Exos could decrease intracellular ROS and provide protection against excessive cell death caused by hypoxia, via secreting extracellular HSP90 by bounding to low-density lipoprotein receptor-related protein 1 (LRP1) and triggering subsequent pathway [103]. Consistent with this, Hsu HH et al. found that ADMSCs-Exos could accelerate the repairment process of diabetic wounds through the stimulation of re-epithelialization, and angiogenesis. Mechanistically, ADMSCs-Exos could induce fibroblast proliferation through the stimulation of TGF-β/Smad3 pathways, which, in turn, stimulates the synthesis of type I collagen in diabetic wounds [112]. Re-epithelialization have been accelerated in the MenSCs-exosomes treated mice as a result of the activation of NF-κB pathways and elevation of NF-κB p65 subunit [95].

The treatment strategies for nonhealing diabetic wounds can be optimized by the implementation of stem-cell-based therapy and the injection of innovative pharmacological substances, for instance unctional miRNAs. Lv et al. developed a hADMSCs-Exos-based miRNA delivery method, and loaded miR-21–5p mimics into hADMSCs-Exos by electroporation [106]. They demonstrated that ADMSCs-Exos, combined with miR-21–5p, enhanced keratinocyte migration and improve the reepithelization in diabetic wounds, by up-regulating the MMP7 expression by activating of the Wnt/β-catenin pathway.

MSCs-derived exosomes exhibit potential suitability for therapeutic applications in the management of diabetic wounds and represent a viable avenue for understanding the mechanisms behind MSCs-derived exosomes. Zhao et al. successfully extracted exosomes from gingival mesenchymal stem cells (GMSCs) and subsequently incorporated these exosomes into the chitosan/silk hydrogel sponge. They found that the utilization of hydrogel contained GMSC-derived exosomes shown significant efficacy in facilitating wound repairment in diabetic rats by the process of collagen remodeling, and the augmentation of angiogenesis and neuronal ingrowth [113]. In addition, Wang et al. have successfully manufactured an injectable FHE hydrogel composed of polypeptide (F127/OHA-EPL) integrated with ADMSCs-exosomes that possesses both self-healing and antibacterial capabilities. The hydrogel demonstrated a notable capacity to improve the effectiveness of healing full-thickness cutaneous wounds in subjects with diabetes and showed a notable ability to augment the proliferation, migration and tube formation capabilities of HUVECs in vitro, suggesting its potential for facilitating complete skin regeneration [114]. In the same year, researchers fabricated a pH-responsive exosomes using FEP, an injectable adhesive dressing made of thermosensitive multifunctional polysaccharide. The use of this dressing expedited the healing course of wounds by promoting the angiogenesis process within the wounds tissue [115]. These works provide the novel approaches for repairing persistent wounds completely.

### Diabetic retinopathy

Blindness due to DR, with incidence is estimated to 3.2 million in 2020, is a permanent condition that imposes significant economic costs on both families and society [116]. Due to its classification as a microvascular disease, a range of therapeutic interventions, such as anti-VEGF therapies, laser treatment and vitrectomy, have been used clinically to mitigate the occurrence of diabetes-related retinal neurovascular complications, such as retinal neovascularization and vitreous hemorrhage. Nevertheless, not all patients respond well to anti-VEGF therapies [117]. The positive benefits of MSCs-Exos have been shown in ocular illness models, through ameliorating functional impairment, neuroinflammation, and cell apoptosis [118] (Table 4).

**Table 4:** The role of exosomes derived from MSCs in other diabetic complications.

DM complication	MSC type	Exosomes reactive molecules/ Genetic modification/Biomaterials	Mechanism of action	Effect	Ref
Diabetic retinopathy (DR)	BMSCs	miRNA-486–3p	Downregulate TLR4/NF-kB pathway	Inhibit oxidative stress, inflammation, and apoptosis	[120]
	BMSCs	miRNA-133b-3p	FBN1 repression	Inhibit angiogenesis and oxidative stress	[121]
	BMSCs	lncRNA SNHG7	Interacts with the miR-34a-5p/XBP1 axis	Inhibit tube formation and angiogenesis	[122]
	BMSCs	unspecified	Suppress Wnt/β-catenin pathway	Reduce oxidative stress, inflammation, and angiogenesis	[124]
	hUCMSCs	unspecified	Downregulation of VEGFA	Ameliorate laser-induced retinal injury	[126]
	hUCMSCs	BDNF	Activate the BDNF-TrkB pathway	Ameliorate neuronal cell viability and inhibit apoptosis	[128]
	hUCMSCs	miRNA-17–3p	Target STAT1	Ameliorate retinal inflammation and oxidative damage	[129]
	hUCMSCs	miRNA-126	Inhibit HMGB1 and the inflammasome	Alleviate inflammation and the DR progression	[130]
	ADMSCs	miRNA-222	Protect retinal tissue structure from damage by regulating STAT5A expression	Alleviate the DR progression	[132]
Diabetic cardiomyopathy (DCM)	MSCs	unspecified	Inhibit TGF-β1/Smad2 signaling pathway	Improve DM-induced myocardial injury and fibrosis	[136]
	hUCMSCs	unspecified	Activate the AMPK-ULK1 pathway, reduce the myocardial autophagy	Alleviate cardiac insufficiency and ventricular remodeling	[141]
	BMSCs	miRNA-125	Increase stress resistance of cardiomyocytes and prevent apoptosis through down-regulation of SIRT7	Recover the cardiac function of ischemia reperfusion injury	[134]
Diabetic stroke	BMSCs	miRNA-133b	Enhance neurite remodeling and angiogenesis	Improve neurologic outcome	[144]
	BMSCs	miRNA-9	Target ABCA1 pathway	Promote neurorestorative effects	[145]
	hUCMSCs	miRNA-126	Decrease myocardial cross-sectional area, interstitial fibrosis, and increase myocardial capillary density	Improve cardiac function	[146]
Diabetic cognitive dysfunction	BMSCs	miRNA-145	Activate downstream genes ABCA1 and IGFR1	Increase vascular and white matter remodeling and improved functional outcome	[149]
	BMSCs	unspecified	Recover cognition impairment	Alleviate the DCM progression	[150]
	BMSCs	unspecified	Increase synaptic density, and improve ultrastructural abnormalities	Recover cognition impairment and histologic abnormity	[151]
	BMSCs	miRNA-146a	Exert anti-inflammatory effects and prevent diabetes-induced cognitive impairment	Ameliorate cognitive impairment	[152]
Diabetic osteoporosis and bone-fat imbalance	BMSCs	miRNA-221	Suppress osteogenesis and promote adipogenesis	Promote bone regeneration	[155]
	BMSCs	unspecified	Enhance the angiogenic differentiation of HUVECs and osteogenic activity of BMSCs	Promote bone regeneration and neovascularization	[156]
	BMSCs	miRNA-140–3p	Target the plexin B1/RohA/ROCK signaling pathway	Alleviate bone degradation and promote bone restoration	[157]
	ADMSCs	miRNA-146a	Inactivation of inflammasome, restraint bone resorption	Alleviate diabetic osteoporosis	[128]
Diabetic erectile dysfunction (DED)	ADMSCs	unspecified	Restore the expression of cGMP, nNOS, ANP and BNP	Improve neurovascular function and inhibit inflammatory factors	[162]
	ADMSCs	miRNA‐126, miRNA‐132, miRNA‐130a, miRNA‐let7c miRNA‐let7b	Induce the proliferation of endothelial cells, reduce cavernous fibrosis	Promote angiogenesis and anti-fibrosis	[163]
	ADMSCs	unspecified	Inhibit corpus cavernosum endothelial apoptosis and raise the proportion of intracavernosal pressure to mean arterial pressure	Vascularization and anti-apoptosis	[164]
	Human urine-derived stem cells	miRNA-21–5p, miRNA-148a-3p, let-7 family, miRNA-10 family, miRNA-30 family	Promote smooth muscle cells regeneration, decrease collagen deposition	Promote angiogenesis and ameliorate erectile dysfunction	[165]
	BMSCs	miRNA-21–5p	Reduce apoptosis, promote cavernous smooth muscle cells proliferation through programmed cell death 4 downregulation	Ameliorate erectile dysfunction	[166]
Diabetic peripheral neuropathy (DPN)	BMSCs	miRNA-17, miRNA-23a, miRNA-125b	Suppress TLR4/NF-kB pathway and RAGE signaling	Alleviate neurovascular dysfunction and improve functional recovery	[168]
	BMSCs	Loaded with miRNA-146a	Inhibit TLR4/NF-kB pathway	Suppress pro-inflammatory gene expression	[170]
	BMSCs	Fused with polypyrrole nanoparticles	Enhance neuroprotective and antioxidant effects	Promote nerve regeneration	[171]
Diabetic vascular calcification	BMSCs	miRNA-146a	Target TXNIP/ROS signaling pathway	Inhibit vascular calcification	[174]
Submandibular gland dysfunction	BMSCs	unspecified	Reduce salivary IgA and serum amylase levels by suppressing the TGF-βpathway through Smad2/3	Improve the salivary glands function and reduce fibrosis, degeneration, and apoptosis	[177]

Previous studies have demonstrated that BMSCs-Exos could mitigate inflammation, oxidative stress and apoptosis, as well as facilitate angiogenesis and promote proliferation of retinal cells in DR mice. It is reported that Toll-like receptor (TLR) 4 predisposes DR. Specifically, the overexpression of TLR4 in ECs has been identified as a major factor in the enhanced inflammatory reactions observed in DR, hence contributing to the further pathogenesis of DR [119]. In the most recent study, the effects of BMSCs-Exos in DR were investigated. The elucidated that BMSCs-Exos, transporting miR-486–3p by reducing the activity the TLR4/NF-kB signaling pathway, suppressed apoptosis of retinal cells [120]. Another study also found that exosomal miRNA-133b-3p, originating from BMSCs, inhibits angiogenesis and reduces oxidative stress in DR by repressiong the expression of FBN1 [121]. In addition, BMSCs-Exos containing the lncRNA SNHG7 could effectively impede the process of tubes formation in human retinal microvascular endothelial cells, and have the potential capacity to suppress endothelial-mesenchymal transition and angiogenesis of capillaries in high glucose condition, through interaction with the miR-34a-5p/XBP1 signaling pathway [122]. The Wnt/β-catenin pathway becomes active during pathogenetic process of DR, resulting in the development of inflammation, microvascular injury and retinal vascular leakage [123]. The upregulation of Wnt/β-catenin pathway has been shown in rats of diabetes induced by STZ, with accompanying notable rise in both phosphorylated and total LRP6, which is related with elevated total β-catenin and a decrease in phosphorylated β-catenin [124]. BMSCs-Exos suppressed the Wnt/β-catenin pathway in retinal damage with subsequent decrease of oxidative stress, angiogenesis, and inflammation, hence suggesting a promising therapeutic strategy for DR [124].

The exosomes from hUCMSCs significantly alleviated the disturbance of retinal structure, decreased the apoptosis of retinal ganglion cells and retinal neurodegeneration, through the inhibition of p38/MAPK pathway [125]. A study reported that hUCMSCs-Exos could improve the effects of blue light stimulation on retinal pigment epithelium cells and retinal damage indeced by laser through the downregulation of VEGFA and induce VEGF expression remarkably [126]. A predominant neurotrophic factor, called brain-derived neurotrophic factor (BDNF) that works by attaching to the TrkB receptor and subsequently initiating the activation of the extracellular signal regulated PI3K pathways in the retina [127]. A study revealed that hUCMSCs-Exos had the potential to transport BDNF into retinal neurons in rats, enhancing high glucose-induced neuronal cell ability and blocking neuronal apoptosis by the activation of BDNF-TrkB pathway [128]. In another study, hUCMSCs-Exos were able to shuffle miRNA-17–3p to improve oxidative damage and inflammatory reactions in DR mice through specifically targeting of STAT1 [129]. Additionally, the exosomes derived from hUCMSCs could effectively reverse inflammatory reaction both in vivo and vitro under the conditions of elevated glucose levels. Zhang et al. documented that the administration of MSCs-Exos by intravitreal injection into the vitreous in rats with diabetes resulted in an effective decrease in the expression of inflammatory factors (IL-1β, caspase-1, and IL-18), and successfully suppressing inflammation. High-mobility group box 1 (HMGB1) levels and inflammasome activities in human retinal ECs were demonstrated to be suppressed by exosomes obtained from miR-126-transfected MSCs under the effect of hyperglycemia [130].

**Fig. 4:**
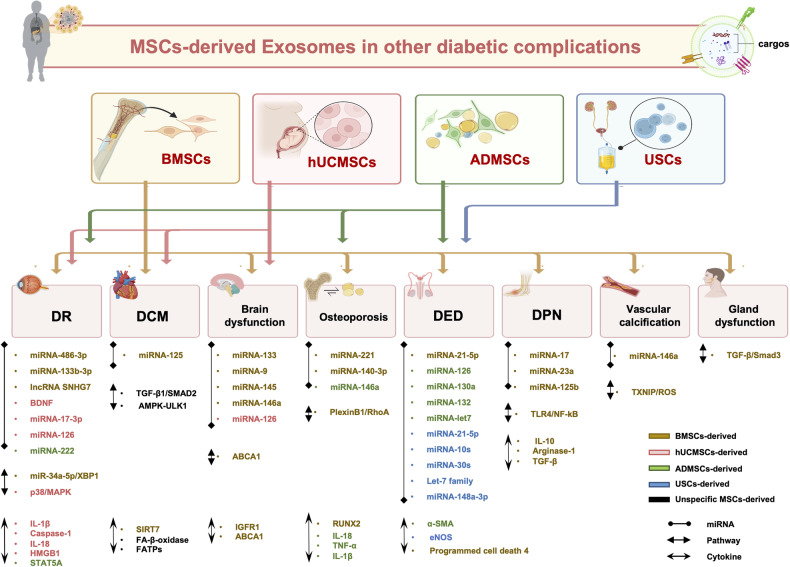
The role of MSCs-derived exosomes in diabetic complications. Exosomes from different MSCs regulate diabetic complications, including DR, DCM, DPN, vascular calcification, and skeletal, neurological, and reproductive degeneration by targeting multiple signaling pathways. This is mainly achieved through their secreted components, which include various miRNAs and cytokines.

Furthermore, a research conducted by Safwat et al. injected ADMSCs-Exos by various routes (intravenous, subconjunctival, and intraocular) into diabetic rabbits and demonstrated that, except intravenous injection, both subconjunctival and intraocular administration of ADMSCs-Exos exhibited protective effects on retinal tissue structure and ameliorate the progression of DR. The authors also demonstrated MSCs-derived exosomes mediated transfer of miR-222 leading to the regenerative alterations of retina tissue [131]. The expression of miR-222 suppressed excessive angiogenesis closely linked to the severity of DR by the regulatory role on STAT5A protein [132]. As shown in Fig. 4, development of MSCs-derived exosome should possibly have great potential in the therapeutics of DR.

### Diabetic cardiomyopathy

Pathologically, DCM is associated with the presence of endothelial dysfunction, myocyte hypertrophy, as well as necrosis and apoptosis. SIRT7, a member of the mammalian sirtuin family, recognized for its substantial influence on the modulation stress response and cell death within the heart [133]. It is observed that BMSCs-Exos carrying miR-125 could recover the heart function in rats afflicted with ischemia reperfusion damage through reducing SIRT7 expression. Consequently, BMSCs-Exos carrying miR-125 exhibited promise as a viable therapeutic intervention for myocardial ischemia reperfusion damage [134] (Table 4).

Cardiac remodeling often manifests in the early phase of cardiomyopathy associated with diabetes, which mostly leads to left ventricular diastolic dysfunction [135]. As a consequence, the potential efficacy of inhibiting ventricular remodeling in enhancing cardiac function is worth considering. In the diabetic rats, there is a notable rise in left ventricular collagen (LVC), accompanied by a significantly decreased in myocardial tissue lipid metabolism related enzymes. A study by Lin et al. administered MSCs-exosomes to rats with diabetes induced by STZ once a week for 12 weeks, and indicated that MSCs-Exos could restore the elevated level of LVC and reduce the fatty acid beta oxidase (FA-β-oxidase) and fatty acid transporters (FATPs) expression. Then researchers also demonstrated that MSCs-Exos improve DM-induced myocardial fibrosis and damage through the suppression of TGF-β1/SMAD2 signaling pathway in mechanistic analysis [136]. The functions of cardiomyocyte autophagy may have conflicting conclusion in DCM [137–140]. The exosomes derived from hUCMSCs were reported to improve cardiac insufficiency and ventricular remodeling, through suppressing excessive autophagy mediated by the AMPK-ULK1 pathway in DCM rats [141]. These findings offer empirical support for the therapeutic efficacy of MSCs-exosomes in the management of DCM (Fig. 4).

### Diabetic stroke

DM induces an assortment of vascular diseases, which is associated with the higher morbidity observed in cases of ischemic stroke [142]. The exacerbation of vascular injury and the presence of an inflammatory milieu likely contributes to unfavorable outcomes following a stroke [142]. The therapies that benefit stroke patients without diabetes may not always be applicable to diabetic stroke patients [143]. Hence, it is imperative to develop treatment strategies for stroke in individuals with diabetes. MSCs‐exosomes contain increased miRNA (miR-133b) treatment boosts the restoration of normal function, promotes neurite remodeling, stimulates the generation of new neurons, and facilitates angiogenesis in the brain affected by ischemic [144] (Table 4).

The BMSCs exosomes were used to treat T2DM rats after 3 days of stroke and found that exosomes derived from BMSCs could decrease blood brain barrier (BBB) permeability and hemorrhage, promote neurorestorative processes, improve long‐term neurological function, and alleviate post-stroke weight reduction. Specifically, the partial contribution of anti-inflammatory responses and white matter remodeling caused by BMSCs-Exos treatment may be attributed to the downregulation of miR-9 expression and the rise in adenosine triphosphatebinding cassette transporter 1 (ABCA1) pathway [145]. In another study, Venkat et al. documented that administration of CD133^+^ human-cord-blood-derivedstem cells derived exosomes might result in a reduction in interstitial fibrosis, myocardial cross-sectional area, quantities of M1 macrophages, NADPH oxidase 2 (NOX2) in cardiac tissue. Additionally, the treatment led to a downregulation of TGF-β, while upregulate the levels of miRNA-126 in the cardiac tissue of stroke mice induced by T2DM [146]. These findings indicated that MSCs-exosomes have promise as a promising and potential therapeutic intervention for managing stroke in subjects with diabetes (Table 4 and Fig. 4).

### Diabetic cognitive dysfunction

Diabetic cognitive dysfunction is characterized by deficits in memory, executive function, attention, processing speed. T2DM is associated with around twofold rise in the risk of senile dementia, but there is still no effective therapeutic method [147]. Recently, as shown in Table 4, some MSCs-derived exosomes were identified have potential for enhancing cognitive dysfunction in diabetic patients.

Within 24-h timeframe of cerebral ischemia, circulating miR-145 significantly increases, which has a significant correlation with raised levels of serum inflammatory factor interleukin-6 [148]. The administration of BMSCs-Exos significantly enhanced vascular growth, white matter remodeling and improved functional outcomes, through decreased miR-145 expression, which activating downstream genes insulin-like growth factor 1 receptor (IGFR1) and ABCA1 in T1DM stroke rats [149]. Zhao et al. found that diabetes mouse, which were injected exosomes from BMSCs through intracranial injection, exhibited a reduced escape latency in a water-maze experiment and ameliorated cognitive dysfunction [150]. Similarly, Nakano et al. observed that injected BMSCs-Exos were taken up by both astrocytes and neurons, subsequently reversed the cognitive dysfunction in STZ diabetic mice, especially the restoration of astrocyte function might enhance neuronal activity, promote brain homeostasis, and facilitate synaptogenesis [151]. Additionally, another study suggested that the secretion of exosomal miRNA-146a by endogenous BMSCs was stimulated up-regulation by enriched environment, and subsequently had anti-inflammatory effects on injured astrocytes as well as served as a preventive measure against diabetes-induced cognitive impairment in STZ-induced diabetic rats [152]. Therefore, exosomes deriving from MSCs can be a new preparation treating diabetic cognitive dysfunction with application prospects (Fig. 4).

### Diabetic osteoporosis and bone-fat imbalance

Accumulating evidence has emerged that a strong correlation between bone metabolism and glucose metabolism [153]. The equilibrium between bone resorption and bone formation was disrupted in diabetes, leading to an increased bone frailty and susceptibility to osteoporotic fractures [154]. Thus, osteoporosis has recently been recognized as a complication of DM, called DM-induced bone fragility [153]. MSCs possess distinctive attributes such as their capacity for multi-directional differentiation and ability to self-renew, which are essential for maintaining the tissue homeostasis. Numerous studies have illustrated the involvement of exosomes in facilitating the impact of MSCs in encouraging osteogenesis and perhaps modulating the process of bone metabolism (Table 4).

Bone-fat imbalance exists in diabetes, which is defined by a lower bone mass and higher marrow fat accumulation [155]. Elevated glucose condition might potentially impact the osteogenic and adipogenic differentiation in MSCs. BMSCs are negatively affected by high glucose condition in DM, including the modifications in the differentiation processes of MSCs, namely osteogenic and adipogenic differentiation, along with the impairment of angiogenic ability [155]. Exosomes released by normal BMSCs exhibited a robust capacity to repress adipogenesis and promote osteogenesis, while these beneficial effects were significantly reduced in BMSCs-Exos obtained from diabetes [155]. miR-221, an abundantly expressed miRNAs in diabetic BMSCs-derived exosomes, has demonstrated the capacity to inhibit osteogenesis and enhance adipogenesis, via downstream target RUNX2. Delayed bone repair in diabetes patients already represents an intractable medical challenge. Intriguingly, the targeted administration of normal BMSCs-Exos to BMSCs utilizing the aptamer delivery method yielded notable outcomes. These included enhanced bone production, decreased deposition of fat in the marrow, and facilitated bone regeneration in mice afflicted with diabetes [155]. Constituent with this, previous study also discovered that exosomes released by normal BMSCs and diabetic BMSCs both exhibited the ability to enhance angiogenic activity of HUVECs and the osteogenic capability of BMSCs. Furthermore, these exosomes were found to promote neovascularization and bone regeneration in calvarial defects rat, but exosomes derived from normal-BMSCs had a more pronounced effect compared to diabetic BMSC-exos [156]. Similarly, exosomes both delivered by normal BMSCs and diabetic BMSCs promoted osteoblastogenesis and mineralization, and augmented skeletal regeneration in a femoral defect model either in normal rats or diabetic rats [157]. Additionally, compared with diabetes mellitus-exosomes, normal-BMSC-exos, highly expressed miR-140–3p, accelerated the repairment process of diabetic wounds by facilitating the osteogenic differentiation of BMSCs through the suppression of plexin B1 which serves as the Sema4D receptor and is involved in the plexin B1/RhoA/ROCK pathways [157]. Inflammation conditions are recognized as prominent contributors in the development of diabetic osteoporosis. Exosomes secreted by ADMSCs shown a potential to suppress inflammation. ADMSCs-Exos had the ability to suppress the synthesis of proinflammatory cytokines (IL-1β, IL-18) in osteoclasts exposed to high glucose, reduce the bone resorption and restore bone loss through the deactivation of NLRP3 inflammasome in osteoclasts [158]. The miR-146a exerted a significant function in the anti-inflammatory impact of exosomes. The concurrent application of ADMSCs-Exos and miR-146a demonstrated a heightened efficacy in suppressing the synthesis of pro-inflammatory cytokines, such as IL-18, TNF-α and IL-1β, in osteoclasts exposed to high glucose conditions. Additionally, this combination treatment could lead to the inactivation of inflammasome, inhibition of bone resorption, whereafter recovery of the bone loss in rats afflicted with STZ-induced diabetic osteoporosis [158]. These offers a new insight and a promising cell-free therapeutic approach for treating diabetic bone-fat imbalance, bone loss and bone unhealing (Table 4 and Fig. 4).

### Erectile dysfunction

More than half of men with DM have erectile dysfunction (ED) worldwide, with a prevalence 3.5‐fold times higher than those without DM [159]. Diabetic erectile dysfunction (DED) involves multiple pathogenesis, such as the dysregulation of VEGF signaling transduction due to hyperglycemia, elevated levels of advanced glycation end products (AGEs), the synthesis of nitric oxide synthase (NOS), heightened production of oxygen free radicals in neurons, and reduced NO and cyclic guanosine monophosphate (cGMP)-dependent protein kinase-1 (cGKI) [160]. The deleterious effects of hyperglycemia and endothelial dysfunction of the vascular lining caused by oxidative stress is the basic pathophysiology of DED.

ADMSC-Exos have generated considerable attention as a prospective therapeutic approach for managing DED (Table 4 and Fig. 4). Prior researches have established that the involvement of cGKI and NO pathway is significant in the maintenance of natural erectile function, and that the depletion of cGMP leads to the occurrence of ED [161]. ADMSC-exosomes reinstated the expression of cGMP through delivery of the enzyme Corin and triggered brain natriuretic peptide (BNP), atrial natriuretic peptide (ANP) and neuronal NOS in diabetic rats [162]. Additionally, the inflammatory cytokines expression in cavernous tissues was suppressed. The aforementioned intervention promoted neurovascular functionality and suppressed the synthesis of inflammatory cytokines, hence counteracting the progression of DED induced by DM.

Diabetes related oxidative stressors lead to the apoptosis of smooth muscle cells and ECs inside the corpus cavernosum, further affecting penile hemodynamics and the development of ED. ADMSC-Exos contain specific miRNAs that have anti-fibrotic properties (miR-let7b and miR-let7c) as well as pro-angiogenic properties (miR-126, miR-132, and miR-130a) (Table 4 and Fig. 4). These exosomes exhibited angiogenic properties and have the capacity to induce the growth of ECs, enhance the expression of alpha-smooth muscle actin (referred to as α-SMA), and promote the production of anti-apoptotic proteins Bcl-2, mitigate cavernous fibrosis, restore erectile function and alleviate ED in diabetic rats [163]. In another study, ADMSCs-Exos administered by intracavernous injection resulted in an elevation in the proportion of intracavernosal pressure to mean arterial pressure noticeably and inhibited apoptosis of smooth muscle cells and ECs, thereby facilitating the restoration of erectile function in rats with DM [164] (Table 4 and Fig. 4). Exosomes derived from USCs have shown promise in addressing DED by ameliorating the functionality of ECs within the corpus cavernosum, through the presence of pro-angiogenic miRNAs, including miR-21–5p, miR-148a-3, miR-30 family, miR-10 family, and let-7 family, which contribute to an upregulation eNOS expression. Furthermore, USCs-Exos can also enhance the neurogenic-mediated erectile response, promote the regeneration of smooth muscle cells inside cavernosum, and reduce collagen deposition in diabetic rats [165] (Fig. 4). Consistent with this, BMSCs-Exos containing miR-21–5p demonstrated a decrease in apoptosis, a promotion of proliferation in cavernous smooth muscle cell, and an improvement in ED in diabetic rats by downregulating programmed cell death 4 [166] (Fig. 4).

### Diabetic peripheral neuropathy

A prevalent chronic consequences of DM, diabetic peripheral neuropathy (DPN), is characterized by the loss of axonal and demyelination [167]. The current focus of DPN therapy is mostly on alleviating symptoms rather than addressing the fundamental pathologic process. Hence, it is imperative to expedite the the progress of developing efficacious therapeutic strategies aimed at improving DPN.

BMSCs-Exos have been shown to mitigate neurovascular dysfunction and axonal demyelination, resulting in improved neurological outcomes [168]. These exosomes significantly resulted in a notable decrease in the threshold for heat and mechanical stimuli, as well as an increase in nerve conduction velocity in mice with diabete. Macrophages have a crucial role as significant regulators of neuroinflammatory, exerting their functions inside the neurovascular system and contributing to the progression of peripheral neuropathy [169]. Treatment with BMSCs-Exos resulted in a substantial decrease number of CD68^+^ macrophages, a downregulation of TNF-α and IL-1β expression, and an elevation in IL-10, arginase-1, TGF-β and polarization towards M2 macrophage. Furthermore, it has been demonstrated that BMSCs-Exos, through delivering of miR-23a, miR-17, and miR-125b, can effectively suppress the receptor for AGEs and Toll-like receptor (TLR)-4/NF-kB pathway, which are known to upregulate the proinflammatory genes and facilitate the transition of M2 macrophages to M1 macrophages, thereby resulting in DPN development [168]. Hence, BMSCs-Exos could attenuate neurovascular damage and promote the restoration of function in DPN. Notably, compared to normal exosomes, the engineered MSC-Exos loaded with miRNA-146a significantly inhibited the presence of inflammatory peripheral blood mononuclear cells and the ECs activation through the suppression of TLR4/NF-κB pathway [170]. Additionally, BMSCs-exosomes, which were combined with polypyrrole nanoparticles encapsulating liposomes, have neuroprotective and antioxidant potential to promote nerve regeneration in DPN rats [171]. These works provide a potential effective approach for managing DPN (Table 4 and Fig. 4).

### Diabetic vascular calcification

Vascular calcification is a prevailing complication in individuals diagnosed with DM, distinguished by the buildup of calcium phosphate in cardiovascular structures [172]. Vascular calcification progresses due in large part to the osteogenic differentiation of vascular smooth muscle cells, especially in individuals with diabetes [173]. Exosomes released correlates with the calcification capacity of vascular smooth muscle cells and enriched in the calcified vasculature, indicating that elevated exosomes release, at sites of vascular damage, could prime the vessel wall to calcify [172]. Thioredoxin-interacting protein (TXNIP) belongs to the the α-arrestin family, and deactivates thioredoxin (Trx), ultimately resulting in increased ROS production and vascular inflammation in high glucose circumstance. Secreted exosomes loaded with miRNAs were also demonstrated to regulate the process of vascular calcification in recipient vascular smooth muscle cells [172]. Researchers revealed that BMSCs-Exos contained a substantial concentration of miR-146a. These exosomes were shown to be transferred to VSMCs and effectively suppressed vascular calcification via TXNIP/ROS signaling pathway [174]. Interfering exosomes secretion and altering microRNAs expression might provide potential approaches for the treatment of vascular calcification (Table 4 and Fig. 4).

### Submandibular gland dysfunction

Diabetes is often accompanied by dysfunction of salivary glands, which resulting in the development of xerostomia, a condition characterized by decreased saliva secretion [175]. Submandibular gland dysfunction might be responsible for the heightened vulnerability to oral infections and compromised wound repair in patients with diabetes. TGF-β/Smad3 signals, as a promising target for treating diabetes, plays a cruical role in glucose tolerance and the overall enhancement of metabolic profile [176]. Recently, Abubakr et al. suggested that BMSCs-Exos resulted in the inhibition of TGF-β signaling cascade via Smad2/3 (Fig. 4). The observed suppression led to an enhancement in functionality of the salivary glands, as seen by the decrease in levels of serum amylase and salivary IgA. Additionally, the treatment with BMSCs-Exos was found to mitigate fibrosis, degeneration, and apoptosis in the salivary glands [177]. BMSC-Exos could be an innovative therapeutic approach for addressing diabetic complications involving salivary glands (Table 4).

### Preclinical studies: large animals

STZ-induced diabetic miniature pigs and rhesus and cynomolgus monkeys were wide used preclinical animal models for the investigation of diabetes such as islet transplantation and development of diabetic drugs. More recently, MSCs-based therapies and MSCs secretome have demonstrated potential clinical applications and offer a promising strategy for DM and complications.

Xenotransplantation of porcine islets has been shown to be a viable therapy for type 1 diabetes [178]. However, the islet graft dysfunction caused by hypoxia is a significant obstacle that severely restricts the practicality of islet transplantation [178]. MSC-based treatments have demonstrated efficacy in the prevention of islet destruction and the promotion of prolonged graft survival in various clinical trials [178, 179]. Autologous BMSCs transplantation specifically targeted into the pancreas has demonstrated efficacy in restoring islet functionality, enhancing the function of β-cells, and improving glucose tolerance in miniature pigs with early-stage DM. The transplantation of BMSCs has the potential to reverse high hyperglycemia, leading to a temporary elimination of the need for exogenous insulin treatment and the preservation of blood glucose homeostasis. Moreover, the transplantation of BMSCs may enhance the process of islet repair by facilitating differentiation into new islets and pancreatic ductal epithelial cells, as well as modulating the microcirculation inside the pancreas [180]. Yamada et al. have elucidated that neonatal porcine BMSCs xenotransplantation showed a beneficial therapeutic impact on diabetic wound repairment owing to its ability to stimulate early-stage lymphangiogenesis and angiogenesis through the secretion of multiple growth factors, including TGF-β1, VEGFA, and VEGFC [181].

The human umbilical cord MSCs conditioned medium treatment improved the viability of neonatal porcine islet cell clusters, exhibited inhibitory effects on apoptosis, while promoting autophagic, and elevating the levels of phosphorylated Akt and PI3K class III [182]. Furthermore, it was observed that this conditioned medium reduced the proportion of phosphorylated mammalian target of rapamycin (mTOR) to total mTOR under hypoxic conditions. Additionally, Exosomes derived from hUCMSCs protected neonatal porcine islet cell clusters from the malfunction caused by hypoxia and played a significant role in enhancing resistance to hypoxia through, indicating a promising approach to enhance the outcomes of islet transplantation [183]. Prolonged hyperglycemia induces a range of physiological reactions in the kidneys, collectively referred to as renal stress responses. Clinically, it has been observed that episodes of acute kidney injury (AKI) in individuals with DM are associated with an increased cumulative probability of progressing to end-stage chronic kidney disease [184]. In a recent study, the development of DM was initiated by the implementation of subtotal pancreatectomy and the subsequent administration of a 60 mg/kg dose of STZ in a cynomolgus monkey (Macaca fascicularis) model. This study has shown that d the presence of diabetes exacerbates the severity of renal ischemia-reperfusion injury produced AKI in comparison to a control group without diabetes. Notably, human BMSCs treatment apparently showed a clear correlation with the regeneration of tubular structures within a timeframe of 24 to 48 h and resulted in a notable decrease of the AKI marker known as Ngal, therefore effectively mitigating the occurrence of renal ischemia-reperfusion injury in diabetic monkey [185]. Together, it is possible that MSCs and MSCs-Exos can be used to treat the DM and complications. Nevertheless, several crucial aspects still need to be taken into account about preclinical experiments, including heightened vigilance of cellular immunity, determination of optimal tissue source of MSCs and exosomes, the migratory capabilities of MSCs and exosomes to the specific site, and the optimization of administration routes and culture conditions.

## Challenges and prospects

MSCs-derived exosomes, with the innate ability to transport genetic material, have exhibited promising clinical translational prospects in the management of DM and its complications. Clinical applications of exosomes derived from MSCs continue to highlight various practical obstacles and challenges, including critical technological factors, as well as the assessment of potential adverse effects, such as the biodistribution of MSCs-derived exosomes, fate of injected cells, risk of microthrombosis due to MSCs and MSCs-derived exosomes injection and some others. A comprehensive understanding of the function of MSCs-based treatment in regenerative medicine is still necessary to address the existing disparities between experimental results and real outcomes of clinical trials. Further investigations are needed to illustrate the composition, mechanistic and specific therapeutics of exosomes from different cells or tissues.

## Conclusions

Diabetes represents a persistent medical condition that severely threatens public health worldwide. MSCs-exosomes have the advantageous characteristics of minimal immunogenicity, low probability of tumor transformation, limited aberrant differentiation, high resilience, and the capacity to exert both local and systemic effects, making them promising for current cell-based alternative therapies. This study thoroughly discussed the potential functions of MSCs-Exos in DM and its associated complications minutely, suggesting that MSCs-Exos may serve as a promising and novel cell-free therapy for addressing DM and its complications in the immediate future.

## Data Availability

The data that support the findings of this study are available from the corresponding author upon reasonable request.
